# Analysis of Socioeconomic, Utilization of Maternal Health Services, and Toddler’s Characteristics as Stunting Risk Factors

**DOI:** 10.3390/nu14204373

**Published:** 2022-10-18

**Authors:** Meitria Syahadatina Noor, Meilla Dwi Andrestian, Resa Ana Dina, Ayunina Rizky Ferdina, Zulfiana Dewi, Niken Widyastuti Hariati, Purnawati Hustina Rachman, Muhammad Irwan Setiawan, Windy Tri Yuana, Ali Khomsan

**Affiliations:** 1 Faculty of Medicine, University of Lambung Mangkurat, Banjarmasin 70714, South Kalimantan, Indonesia; 2 Department of Nutrition, Polytechnic of Health Ministry of Health, Banjarbaru 70714, South Kalimantan, Indonesia; 3 Department of Community Nutrition, Faculty of Human Ecology, IPB University, Bogor 16680, West Java, Indonesia; 4 National Research and Innovation Agency, Bogor 16915, West Jawa, Indonesia

**Keywords:** stunting, sociodemographic, maternal health, child’s characteristics, nutritional status

## Abstract

Stunting prevalence in South Kalimantan has been higher than the national figure and is the sixth highest in Indonesia. Not many studies in South Kalimantan have analysed the risk factors for stunting comprehensively that combine sociodemographic factors, utilization of maternal health services, and characteristics of children. Therefore, the purpose of this study was to analyse sociodemographic factors, utilization of maternal health services, and characteristics of children under 5 as determinants of stunting in South Kalimantan Province. This study used an analytic observational method with a cross-sectional design. Data collection used secondary data from the results of South Kalimantan Baseline Health Research 2018. The total population of toddlers obtained from South Kalimantan Baseline Health Research 2018 data was 1218 toddlers, and all of them were taken as samples. Data analysis used a chi square test for bivariate test and Logistic Regression for multivariate test. There is a relationship between mother’s education level (*p* = 0.001), father’s education (*p* = 0.002), toddler age (*p* < 0.001), low birth weight (*p* = 0.05), exclusive breastfeeding (*p* = 0.008), and underweight (*p* = 0.000) with stunting. The data were continued with the Logistics Regression test and the dominant variables related to stunting were underweight (*p* < 0.001 with OR 18,241), under-five age (*p* < 0.001, with OR value for ages 24–35 months 9511), and premature birth (*p* = 0.027 with an OR of 2187). The conclusion of this study is that the most important factor in the incidence of stunting in South Kalimantan is underweight nutritional status.

## 1. Introduction

Stunting in Indonesia is still a nutritional problem that needs serious treatment. The Indonesian Ministry of Health’s data and information centre (2018) states that stunting is a condition in children who have shorter body length/height compared to their peers. This category is based on WHO child growth standards. Stunting is the impaired growth and development that children experience from poor nutrition, repeated infection, and inadequate psychosocial stimulation. Children are defined as stunted if their height-for-age is more than two standard deviations below the WHO Child Growth Standards median. Stunting adversely affects both short- and long-term consequences, including poor child growth and development. The WHO states Indonesia as the country with the third-highest stunting prevalence in Southeast Asia, with a prevalence of 36.4% from 2005 to 2017 [1,2].

The Indonesia Nutrition Status Survey in 2021 reports that the stunting prevalence in Indonesia is 24.4%, while the stunting prevalence target for 2024 is 14% [3]. South Kalimantan is one of the provinces that has a stunting prevalence higher than the national figure, which is 30%. South Kalimantan is ranked sixth highest in Indonesia. The three districts with the highest prevalence of stunting in South Kalimantan are Banjar District (40.2%), Tapin District (33.5%), and Barito Kuala (32.4%) [4]. Wicaksono and Hartanti (2020) explained that the highest incidence of stunting was found at the age of less than 5 years, and 18% of them were classified as severe stunting [5].

Stunting can interfere with the physical and cognitive development of children [1]. In addition, stunting also increases the risk of child mortality, developmental disorders and learning abilities, the risk of suffering from infectious and non-infectious diseases, and reduces productivity and economic ability [5]. Therefore, the handling of stunting must be a priority to improve the quality of Indonesia’s human resources.

Research by Wicaksono and Hartanti (2020) found that the factors associated with the incidence of stunting were individual, household, and community factors. Individual factors consist of gender and immunization status. Household factors consist of the educational status of the father and mother, household welfare, and slum settlements. In addition, there are also factors of influence from the community, namely the place of residence (village or city) [5].

Similar research has also been conducted by Ardiansyah et al. (2018) which states that stunting is caused by age, gender, parental education, parental income, number of household members, early initiation of breastfeeding, exclusive breastfeeding, and maternal height. The results of multivariate analysis showed that the most dominant factor was father’s education [6].

Adolescent pregnancy is also associated with the incidence of stunting as in the research of Larasati et al. (2018). This study shows that the younger a person’s gestational age will increase the incidence of stunting [7]. Until now, there are still not many studies in South Kalimantan that examine the risk factors for stunting in a comprehensive manner that combines individual, household, community, and cultural factors. Thus, this research needs to be carried out.

## 2. Materials and Methods

### 2.1. Study Design and Data Dource

This study used observational analytic with a cross-sectional design with secondary data from the 2018 Baseline Health Research (Riskesdas, acronym in Indonesian). The population of this study was 1218 children aged 0–59 months who were included as the 2018 Baseline Health Research sample in South Kalimantan Province. The secondary analysis for this research was conducted from May to August 2022. Children less than 5 years old in this research were called ‘toddler’.

### 2.2. Ethical Approval

For the primary data collection of Riskesdas 2018, the Ethical Committee of Health Research, NIHRD, the Ministry of Health of Indonesia gave their approval with the reference number LB.02.01/2/KE.267/2017. All participants were asked for their consent and signed the informed consent form in the survey.

### 2.3. Variables

The dependent variable of the study was the incidence of stunting, which is characterized by height-for-age less than -2 SD from the WHO child growth standard. The independent variables of the study were classified into sociodemographic characteristics, utilization of maternal health services, and toddler’s characteristics.

Sociodemographic data as well as history of pregnancy, delivery, and toddler’s feeding practices were collected by trained interviewers with a validated questionnaires. Occupational status of each of the parents were classified into working and not working. Classification of the area of residence into rural and urban follows the classification from Statistics Indonesia, which is the official statistics agency in the country. Household members were numbers of people belonging to the subject’s household.

Variables regarding the utilization of maternal health services include Ante Natal Care (ANC) visits, consumed Iron and Folic Acid (IFA) tablets, childbirth location, and birth attendants. ANC visits were defined as the number of antenatal visits during pregnancy. Consumed IFA tablets refer to the number of IFA supplement tablets consumed by the mother during pregnancy of the child(ren) under 5 years old. A question on childbirth location was asked to know whether or not the child was born in a healthcare facility. Meanwhile, birth attendants of the child were classified into skilled or healthcare worker and non-healthcare worker.

Toddler’s characteristics include age, gender, gestational age, birthweight, immunization status, status of prelacteal feeding, exclusive breastfeeding, underweight, wasting, and age of mother when giving birth to the child. Data from the maternal and child health book (KIA, acronym in Indonesian) were used to observe the child immunization status. The anthropometric measurements were performed by trained enumerators. Premature birth was defined if the gestational age at delivery was less than 20 weeks. Low birth weight was defined when the birth weight of the baby was less than 2500 g. Prelacteal feeding refers to the presence of any other food/drink given to the baby before starting to be breastfed. Additionally, status of adolescent mother is defined if the mother age when giving birth to the baby was 19 years old or younger.

### 2.4. Statistical Analysis

Univariate analysis was performed by looking at the distribution and percentage of each variable. Bivariate analysis was conducted by looking at the relationship of each independent variables with the dependent variable (chi square test with 95% confidence interval). Variables having significant relationships with the stunting status were then entered into the next stage which was the multivariate analysis. Those analyses were performed with a complex samples design using SPSS Version 25.

## 3. Results

### 3.1. The Relationship between Sociodemographic Characteristics, Utilization of Maternal Health Services, and Child Characteristics with the Prevalence of Stunting in South Kalimantan

Bivariate analysis (Table 1) was carried out on all children under 5 who were the samples of the 2018 Baseline Health Research in South Kalimantan. From 1218 children under 5, it was shown that both fathers and mothers who work may increase the risk of stunting (OR: 1076; 95%CI: 0.778–1.489 for mothers and OR: 2747; 95% CI: 0.635–11.885 for fathers). However, the relationship was not statistically significant. On the other hand, there was a significant relationship between mother (*p* 0.001) and father (*p* 0.002) education and stunting in children under 5. Being born to parents who have completed high school can reduce the odds of stunting. The risk of stunting was almost the same in both urban and rural residents as well as in households with less and more than four members.

The results of bivariate analysis showed that babies born in healthcare facilities had a lower risk (OR = 0.788; 95% CI: 0.539–1.153) of being stunted than those whose birth locations were not in health facilities. However, there is no statistically significant relationship between all of the variables about the utilization of maternal health services to the risk of child stunting in this population.

Toddler’s age is a factor that may determine stunting status (*p*-value < 0.001), as can be seen from the large OR value shown in the age category. This risk tends to increase until the toddler is aged 24–35 months, and then decreases again in toddlers aged 36–60 months.

Another variable that was statistically significant in influencing stunting was the status of low birth weight (*p*-value 0.05). Babies whose birth weight were less than 2500 g have a 2.1 times risk of becoming stunted toddlers than babies with normal birth weight (≥ 2500 g).

The variable of exclusive breastfeeding history was associated with the incidence of stunting (*p* = 0.008), but those who received exclusive breastmilk were still at risk of suffering from stunting.

Another variable that is statistically significant is underweight status. Children under 5 who were underweight had a risk of 7.119 times higher of becoming stunted compared to other children under 5.

Other variables such as gender, history of premature birth, immunization status, history of prelacteal intake, wasting status, and being born to adolescent mothers were not statistically associated with stunting in children under 5 years old in South Kalimantan.

Furthermore, from the sociodemographic aspects analysed, the variables of father’s employment status, father’s education level, mother’s education level, place of delivery, age of toddlers, history of low birth weight, history of exclusive breastfeeding, stunting, history of premature birth, and wasting nutritional status were selected as candidates. variables to be analysed are multivariate to obtain the right model (fit model).

### 3.2. Determinants of Stunting in Toddlers in South Kalimantan Kalimantan

The multivariate analysis conducted in this study resulted in four variables related to the incidence of stunting in children under five in South Kalimantan, namely age range, history of premature birth, as well as status of underweight and wasted. These results were obtained after adjusting for other variables, namely the mother’s age at delivery, the father’s education level, and a history of low birth weight.

The underweight status is the main determinant of the incidence of stunting in children under 5 in South Kalimantan with an OR value of 18.241 (95% CI 8.054–41.312).

The age of the toddler is also a determining factor in the incidence of stunting, where the risk begins to appear after the toddler is over the age of 6 months. After the age of 6 months, toddlers begin to be given complementary foods and the process of parenting related to diet becomes very influential on the nutritional status of children. Thus, it can be concluded that the incidence of stunting in the province of South Kalimantan is more influenced by factors of parenting and quality of complementary feeding. The trend of increasing risk was seen in toddler aged 6–11 months (OR 2.688, 95% CI 0.849–8.510), then increased in later toddler and the risk was highest in toddler aged 24–35 months (OR 9.511, 95% CI 3.322–27.234). Furthermore, the risk began to decrease in toddler aged 36–59 months.

Another determinant of stunting was a history of preterm birth (OR 2.187, 95% CI 1.082–4.380). Toddlers with a history of premature birth have a higher risk of stunting than toddlers born at term. However, the wasting variable showed OR 0.129 (95% CI 0.049–0.339). Wasting is a condition when a toddler is underweight in relation to his or her height. A thin toddler has a low body weight but is quite tall. The R-square value obtained is 24.5%.

Thus, it can be interpreted that the incidence of stunting in children under five in South Kalimantan can be explained by 24.5% because of the determinants of underweight, toddler’s age, history of premature birth, and wasting. Meanwhile, 76.5% of stunting risk among this population is influenced by other variables.

## 4. Discussion

### 4.1. Relationship between Sociodemographic Characteristics and Prevalence of Stunting

The results of the study in Table 1 show that there is a significant relationship between the education of mothers and fathers with stunting in children under 5. Parents who have graduated from high school have almost twice the risk of stunting compared to parents who did not complete formal education up to that level.

Education can stimulate nutritional status both directly and indirectly through influencing the awareness and cultural preferences that may shape the habit of food consumption. Ultimately, good dietary habit leads to good nutritional status. Education also affects hygiene and sanitation including sources of drinking water, drainage system, and housing conditions. Good hygiene and sanitation practices will build health by decreasing infection cases [8].

The level of education can play a role in understanding health problems that will have a major impact on nutritional status, in this case stunting. A person’s level of education will affect family income. The higher the level of education, the better the family income, so that it will have an impact on family food security. In addition, the level of parental education is also assumed to affect the level of knowledge, including knowledge about family health and nutrition. This will increase efforts in parenting for children, utilization of health services, sanitation hygiene, and other behaviours [5].

This study is in line with the research of Wicaksono and Hartanti (2020) which states that the education of fathers and mothers is related to the incidence of stunting (*p* value < 0.01), with a minimum high school education can reduce the risk of stunting [4]. Das and Gulshan’s research (2017) also shows the same thing, where the education of fathers and mothers is associated with the incidence of stunting with a *p* value of 0.000 [9].

Other sociodemographic variables in this study, such as father and mother’s employment status, area of residence, and number of family members, were not associated with stunting. Employment is not related to stunting because the identification of work in this study is still common which is not supported by the amount of family income. The limited data on job descriptions causes the description of this type of work to not be able to describe family welfare.

The number of family members is theoretically assumed to affect stunting because it is related to meeting family needs. However, the number of family members in the study was not related to stunting, because the number of family members of children under five who experienced stunting was dominated by <4 people (60.7%), so there were other factors that caused stunting besides family members.

The characteristics of the area of residence (rural and urban) are not a determinant of the cause of stunting in this study.

### 4.2. Relationship between Utilization of Maternal Health Services and Prevalence of Stunting

The variables of the use of maternal health services in this study were ANC visits, consumption of IFA tablets during pregnancy, delivery in health facilities, and birth attendants. All of these variables are not related to the incidence of stunting in the results of the bivariate statistical analysis of this study. However, descriptive data still illustrate the tendency to cause stunting. The descriptive data in Table 1 show that mothers who performed ANC <4 times during pregnancy had 91.9% of stunting prevalence in toddlers.

Health services for pregnant women (ANC) are crucial for the health of the mother and her womb where it was found that mothers who had ANC visits less than four times during pregnancy were more likely to have stunted children at the age of 0–23 months compared to mothers who made ANC visits as many as four times or more [10].

The antenatal phase is an important period to prevent stunting. In this phase, foetal growth occurs and is the optimal period for child development up to the first 1000 days of life. Environmental and nutritional factors in this phase will affect foetal growth, brain development, gastrointestinal tract, metabolism, and immune system. Nutrient intake is very important to support this phase of the first 1000 days of life, including amino acids, iron, iodine, calcium, zinc, magnesium, and vitamins [11].

Based on this explanation, the results of this study which show that ANC is not associated with stunting can be caused by maternal nutritional intake during pregnancy which is the main factor. The nutritional intake was not identified in this study, so even though ANC was carried out, if the nutritional intake was not good, it would still increase the risk of stunting.

Number of consumed IFA supplement tablets during pregnancy was also not associated with the incidence of stunting in this study. However, Table 1 shows that 52.8% mothers who did not take IFA supplement tablets during pregnancy had stunted children.

Consumption of IFA supplement tablets during pregnancy has a linear correlation with child growth. However, in general, growth failure in toddlers is influenced by lack of nutrient intake. In addition to iron, this includes folic acid, calcium, amino acids, and other nutrients [10,11]. Consumption of IFA supplement tablets during pregnancy affects foetal growth because it is associated with anaemia in pregnant women. Anaemia in pregnancy can cause a decrease in the flow of oxygen and nutrients in the placental tissue which will have an impact on disrupting the nutritional status of the foetus [12].

Other nutrients beside iron and folic acid were not identified in this study. Because the IFA supplementation is not the only one that affects the incidence of stunting, the factor of consuming IFA tablets during pregnancy is not associated with the incidence of stunting in this study. In addition, the mechanism that causes growth disorders is the condition of anaemia in pregnant women, where Hb levels were not identified in the study so that it has not been able to describe the condition of anaemia as the cause of growth disorders.

In this study, childbirth location and presence of skilled birth attendant(s) was not associated with stunting. This is shown by mothers who gave birth in health facilities having stunted children by 73.4% (Table 1). Torlesse et al. (2016) explained the ability of medical personnel in providing ANC services and assisting deliveries in health facilities related to stunting [9]. Giving birth in health facilities and birth attendants as risk factors for stunting related to the handling of babies born, such as babies with low birth weight, neonatal examinations, and handling complications during childbirth [12].

This unrelated variable can be assumed because the location of delivery and birth attendants are related to the management for delivery assistance, so there is still an advanced phase that has a long impact on the occurrence of stunting, namely nutritional intake in toddlers.

### 4.3. The Relationship between Child Characteristics and the Prevalence of Stunting

This study analysed several characteristics in children under five that were associated with stunting. Variables that were significantly related were age, birth weight, exclusive breastfeeding, and underweight.

Toddler’s age is a determinant of stunting, as can be seen from the large OR value shown in the toddler age category (Table 1). Children aged 6–11 months have 1.8 times the risk of stunting compared to children aged 0–5 months. This risk tendency increases until children are aged 24–35 months and then decreases again in children aged 36–60 months. Toddlers who are ready to receive supplementary breastfeeding, which is above 6 months, begin to show an increased risk for stunting.

Toddler’s age is a risk factor for stunting because it is related to the growth period. We found that toddlers aged > 6 months have a higher risk of stunting than children aged < 6 months. At this age, they having experienced interactions with environmental factors and feeding patterns [13]. Children aged 0–6 months only receive breast milk, while children aged > 6 months receive complementary foods other than breast milk that is still being given. This illustrates that complementary feeding plays an important role in meeting the nutritional needs of children, especially for their growth and development [9].

This study is in line with Mulu et al. (2022) who explained that age is related to the incidence of stunting [13]. Das and Gulshan’s research (2017) also states the same thing with *p* value = 0.000, where age < 6 months has a risk of becoming stunted by 14–22%, which then along with increasing age also increases the risk of stunting [9].

Another variable that was statistically significant in influencing stunting was low birth weight (*p*-value 0.05). Babies whose birth weight is less than 2500 g have a 2.1-fold risk of becoming stunted compared to babies with normal birth weight (≥2500 g).

Low birth weight (<2500 g) will have an impact in the first 6 months of life. Then the impact will decrease until the child is 24 months old. If the baby can achieve appropriate growth in the first 6 months, then it is possible that the baby has a normal body length. A history of low birth weight indicates growth retardation in the womb that occurs acutely and chronically [14].

This study is in line with the research of Lestari, Hasanah, and Nugroho (2018), which states that low birth weight has a *p* value of 0.006 associated with stunting. Low birth weight increases the risk of stunting by 12,429 times compared to normal birth weight [14].

Exclusive breastfeeding is also a determinant factor for stunting, although we found more stunting cases from children who did not receive exclusive breastfeeding. Our data suggest that the period after 6 months of the completion of exclusive breastfeeding can play a crucial role, especially in terms of the child’s dietary intake. Theoretically, exclusive breastfeeding reduces the risk of stunting. Introducing liquid/solid foods other than breast milk to children aged < 4 months will increase the risk of gastrointestinal disease, which will result in growth disorders, nutritional deficiencies, and susceptibility to infectious diseases until the age of 2 years. Exposure to infectious diseases such as diarrhoea and fever can increase the risk of stunting, where the incidence of this disease is more commonly found in children who are not exclusively breastfed [15].

Early initiation of breastfeeding is also necessary to increase the success of exclusive breastfeeding. The first milk that issues during breastfeeding contains colostrum which is rich in nutrients and antibodies. Colostrum is essential for the growth of the gut microbiota and the immune system. Colostrum is only released in the first 2–3 days after delivery [15].

Our findings about exposure to prelacteal feeding are in line with Kuchenbeker et al. (2015) who shows that early initiation of breastfeeding as one of the practices of exclusive breastfeeding is significantly related to the growth of toddlers. Giving food before the age of 6 months also has an impact on infant growth retardation [15].

Another variable that is statistically significant is underweight. We found that underweight in children under 5 years olds can increase the risk of stunting up to 18.241 times. Underweight conditions can be caused by birth weight, birth length, frequency and quality of maternal ANC, and quality of food consumed by children. Dietary intake of the children must provide sufficient nutrients for their growth and development. However, a culture of dietary restrictions or abstinence can hinder the fulfilment of children’s nutritional needs [16].

The research of Syeda et al. (2021) reported similar finding, where underweight in the second and third years will increase the risk of stunting greater than the age of <1 year. These data are related to the diet given to children by parents [16].

Other characteristics of children under 5 such as gender, history of premature birth, history of prelacteal feeding, nutritional wasting status, and status adolescent mothers were not statistically associated with the incidence of stunting in children under five in South Kalimantan. These data show that stunting can occur in both male and female sexes so that gender differences in parenting patterns can begin to change.

In addition, immunization status is not related to stunting because immunization is already a mandatory program that has been implemented by population, so that it has been received by both stunted and non-stunted toddlers. This is shown by the data in Table 1, where a large a majority of our study subjects have been immunized.

Wasting is also not related to stunting in this study, because weight in wasting is a short-term (acute) nutrition indicator, thus it can change quickly. While stunting has length/height as a long-term (chronic) nutrition indicator, accordingly the factors that influence stunting should already pass on long-lasting impact.

In this study, being born to an adolescent mother was not a risk factor for stunting. This is possibly due to the fact that the number of adolescent mothers who were sampled was relatively too small. Although not significantly related, descriptive data show that from the 1218 toddlers who were sampled, the percentage of stunting in adolescent mothers was 41.7%, which is around 10% higher than the percentage among adult mothers. This percentage can indicate a trend towards the risk of stunting in adolescent mothers.

Almost half of the female population in South Kalimantan married before the age of 19 years old [17], suggesting the need of interventions targeted at adolescent mothers to prevent related health problems. Mother’s age at marriage will affect the maturity of the reproductive organs. Those who were still too young (<20 years old) do not yet have mature reproductive organs physically and functionally. Consequently, they are not ready for conception, give birth, and breastfeed optimally. If a pregnancy occurs at the mother’s age of <20 years old, the adolescent’s growth will stop because the fulfilment of nutritional intake will be allocated to the foetus. Risk factors that may occur in young pregnant women are nutritional disorders such as anaemia, chronic energy deficiency, the risk of experiencing complications during pregnancy and childbirth such as preeclampsia/eclampsia. Based on these risks, the impact that will be experienced by the foetus/baby being born is growth disorders such as Intra Uterine Growth Retardation, low birth weight, and stunting.

However, our study does not confirm adolescent pregnancy as a risk factor for stunting in South Kalimantan. This study is in line with the research of Fonseka et al. (2022) which states that there is no relationship between maternal age and the incidence of stunting. The most important determinant is nutritional intake during childhood [18].

### 4.4. Dominant Factors Associated with Stunting in South Kalimantan

The results of the multivariate analysis in Table 2 show several variables that are more dominant as risk factors for stunting: toddler age, premature birth, underweight, and wasting. If sorted by risk magnitude, the determinants with the greater risk factor are underweight as the most dominant factor, followed by child age 24–35 months, and premature birth.

Toddler who are underweight, and within age range of 24–35 months old are at higher risk of stunting. These variables are related to child’s dietary intake, while premature birth can be related to mother’s age and health conditions during pregnancy including the maternal nutritional status. Thus, the results of the analysis of this study illustrate that nutritional intake is more dominant in increasing the risk of stunting compared to other variables. Those variables (toddler’s age, history of premature birth, and underweight) are simultaneously associated to stunting risk with the magnitude of the association was 24.5%.

Factors that also affect the incidence of stunting beyond those investigated in our study are sensitive factors, such as agriculture and food security, mental health, child protection, water, and sanitation that come from multisector [19].

The basic factors that must be strengthened are the level of community knowledge and education, government policies, budget sources and leadership patterns, social, economic, and political environmental conditions. These basic factors can act as inputs for stunting management [19].

Stunting can be manifested after chronic undernourishment with causes that are multifactorial. Stunting puts children at high risk of infection with delayed recovery as well as growth retardation [20].

From these basic factors, it will affect food security which consists of the availability, access, and utilization of foods. In addition, parenting and eating patterns at the home level, access and use of health facilities, as well as a healthy and safe environment will also be affected. These factors will play a role in the process of tackling stunting. The expected outcome is that the nutritional status and development of the foetus and child will be optimal [19].

Due to the multifactorial nature of the cause of stunting, both sensitive and specific interventions need to be carried out to tackle the problem. Additionally, management and policy reviews can be strengthened as a basis for linking processes to optimize the outputs [19].

## 5. Conclusions

This study concludes that the educational level of both parents, toddler’s age, birth weight, status of exclusive breastfeeding, and underweight are associated with the increased risk of stunting among toddlers in South Kalimantan, Indonesia. The main contributing factors to the risk of stunting is underweight.

## Figures and Tables

**Table 1:** Bivariate Analysis between Independent Variables and Stunting Prevalence.

Variables	Stunted Toddler (n = 396, N = 1218)				
	N	(%)	OR	95% CI	*p* Value
Household Sociodemographic Characteristics					
Working mother Yes No	172 224	43.4 56.6	1.076 1	0.778–1.489	0.656
Working father Yes No	394 2	99.5 0.5	2.747 1	0.635–11.885	0.158
Mother graduated high school Yes No	126 270	31.7 68.3	0.567 1	0.410–0.784	0.001 *
Father graduated high school Yes No	146 250	36.9 63.1	0.591 1	0.422–0.828	0.002 *
Area of residence Urban Rural	146 249	36.9 63.1	0.939 1	0.670–1.316	0.714
Household numbers <4 people ≥4 people	240 156	60.7 39.3	0.945 1	0.677–1.318	0.738
Utilization of Maternal Health Services					
ANC Visit ≥4 <4	19 377	4.7 91.9	1.068 1	0.518–2.201	0.858
Consumed IFA tablets ≥90 <90	187 209	47.2 52.8	1.038 1	0.735–1.466	0.833
Childbirth location Healthcare facility Not a healthcare facility	291 105	73.4 26.6	0.788 1	0.539–1.153	0.220
Birth attendant Skilled Non-skilled	376 20	95.0 5.0	1.146 1	0.522–2.517	0.734
Toddler’s Characteristics					
Age 0–5 months 6–11 months 12–17 months 18–23 months 24–35 months 36–47 months 48–60 months	14 25 19 76 99 90 74	3.5 6.3 4.7 19.3 25.0 22.7 18.6	1 1.803 2.417 5.240 6.718 5.529 3.755	(0.731–4.442) (0.870–6.713) (2.387–11.504) (3.032–14.886) (2.491–12.276) (1.657–8.511)	<0.001 *
Gender Male Female	177 219	55.5 44.5	0.828 1	(0.589–1.162)	0.274
Premature Birth Yes No	91 305	22.9 77.1	1.450 1	(0.947–2.221)	0.068
Low Birth Weight Yes No	(N = 253) 23 230	9.1 90.9	2.127 1	(0.984–4.598)	0.05 *
Basic immunization Complete Incomplete	(N = 256) 210 46	82.0 18.0	1.250 1	(0.765–2.040)	0.372
Received prelacteal food Yes No	(N = 125) 83 42	66.6 33.4	1.257 1	(0.734–2.152)	0.403
Exclusively breastfed Yes No	(N = 125) 61 64	48.5 51.5	1.984 1	(1.188–3.315)	0.008 *
Underweight Yes No	174 222	56.1 43.9	7.119 1	(4.777–10.610)	0.000 *
Wasted Yes No	40 356	10.0 90.0	0.624 1	(0.365–1.065)	0.082
Mother’s age at birth ≤19 years >19 years	25 371	6.3 93.7	1.539 1	(0.589–2.931)	0.189

Note: * significant at α = 0.05.

**Table 2:** Factors Associated with Stunting in South Kalimantan.

Variable	Stunted Children		
	OR	95% CI	*p* Value
Mother’s age at birth >19 years old ≤19 years old	1.139 1.000	(0.466–2.786)	0.775
Father graduated high school Yes No	0.813 1.000	(0.497–1.328)	0.407
Mother graduated high school Yes No	1.030 1.000	(0.640–1.656)	0.904
Age 0–5 months 6–11 months 12–17 months 18–23 months 24–35 months 35–47 months 48–60 months	1.000 2.688 3.516 7.809 9.511 7.273 5.229	(0.849–8.510) (0.803–15.385) (2.728–22.348) (3.322–27.234) (2.360–22.409) (1.693–16.155)	<0.001 *
Premature Birth Yes No	2.187 1.000	(1.082–4.380)	0.027 *
Low birth weight Yes No	2.093 1.000	(0.792–5.529)	0.136
Underweight Yes No	18.241 1.000	(8.054–41.312)	<0.001 *
Wasted Yes No	0.129 1.000	(0.049–0.339)	<0.001 *

R-Square: 0.245. * significant at α = 0.05.

## Data Availability

The 2018 Indonesian Baseline Health Research data used in this study were supplied by the Health Development Policy Agency of the Indonesian Ministry of Health under license and so cannot be made freely available. Requests for access to these data should be made to the Health Development Policy Agency of the Indonesian Ministry of Health.
